# The Correlation between *Prorocentrum donghaiense* Blooms and the Taiwan Warm Current in the East China Sea - Evidence for the “Pelagic Seed Bank” Hypothesis

**DOI:** 10.1371/journal.pone.0064188

**Published:** 2013-05-09

**Authors:** Xinfeng Dai, Douding Lu, Weibing Guan, Ping Xia, Hongxia Wang, Piaoxia He, Dongsheng Zhang

**Affiliations:** State Key Laboratory of Satellite Ocean Environment Dynamics, The Second Institute of Oceanography, SOA, Hangzhou, China; University of Connecticut, United States of America

## Abstract

During the last two decades, large-scale high biomass algal blooms of the dinoflagellate *Prorocentrum donghaiense* Lu have occurred frequently in the East China Sea (ECS). The role of increasing nutrient concentrations in driving those blooms is well-established, but the source population that initiates them is poorly understood. We hypothesized that the front of Taiwan Warm Current (TWC) may serve as a ‘seed bank’ that initiates *P. donghaiense* blooms in the ECS, as the physiochemical conditions in the TWC are suitable for the growth of *P. donghaiense*. In order to test this hypothesis, two surveys at different spatio-temporal scales were conducted in 2010 and 2011. We found a strong correlation in space and time between the abundance of *P. donghaiense* and the TWC. The spatial extent of the *P. donghaiense* bloom coincided with the TWC front in both 2010 and 2011. During the early development of the blooms, *P. donghaiense* concentration was highest at the TWC front, and then the bloom mass shifted inshore over the course of our 2011 survey. The TWC also moved inshore, albeit after the appearance of *P. donghaiense*. Overall, these results support our hypothesis that *P. donghaiense* blooms develop from the population at the TWC front in the ECS, suggesting the role of the ocean current front as a seed bank to dinoflagellate blooms.

## Introduction

Large-scale high biomass algal blooms of the dinoflagellate *Prorocentrum donghaiense* Lu have occurred frequently in the East China Sea (ECS) over the last two decades. These blooms are massive, sometimes extending over thousands of square kilometers, and can persist for nearly one month [1], [2]. The blooms are considered to be driven by increasing nutrient inputs from the Changjiang River (the largest river in China) and upwelling [3]–[5].

One aspect of the *P. donghaiense* bloom that is poorly understood is its initiation. A hypothesis is that a “pelagic seed bank” of low number of *P. donghaiense* cells offshore may act as an inoculum. The hypothesis, proposed by Smayda (2002), postulates that algal cells that have accumulated at ocean current fronts may act as bloom inoculums in upwelling zones [6]. The proposed mechanism is in contrast to the initiation mechanism that has been described for cyst-forming dinoflagellate species (e.g. *Alexandrium fundyense*) that are inoculated through the germination of their benthic cysts [7], [8]. No such cyst stage has been described for *P. donghaiense* [4]. Therefore its initiation is more likely to rely on mechanisms like the “pelagic seed bank” that deliver vegetative cells to nutrient rich waters like upwelling zones.

There is an upwelling belt in the ECS, about 40 km in width between the 20 m and 50 m isobaths and parallel to the Zhejiang coast line [9]–[11]. The upwelling is mainly induced by the continental slope and the Taiwan Warm Current (TWC) [12], [13]. As a branch of the Kuroshio Current (KC), the TWC is characterized by a high temperature and salinity, making it suitable for the survival and growth of *P. donghaiense* cells even in winter time [14], [15].

**Figure 1:**
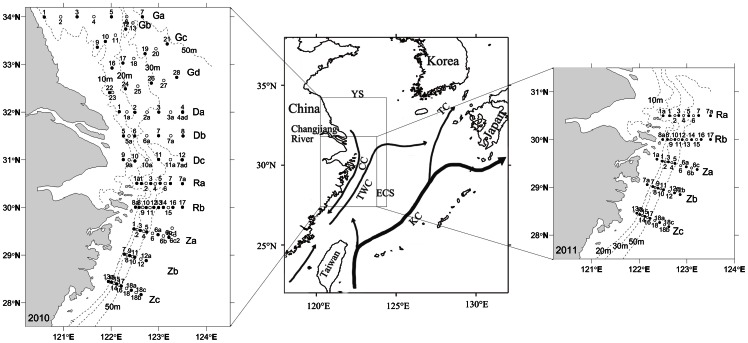
Sampling sites and Circulation pattern in the East China Sea (ECS) (modified from [4], [16]). Left panel and right panel show the station locations in 2010 and 2011, respectively. Closed circle indicates comprehensive stations and open circle indicates hydrological stations, with labels above or below the station symbols, respectively. Transect labels are marked on the right of transects. Dot line indicates the isobath line. Middle panel, YS: Yellow Sea; KC: Kuroshio Current; TWC: Taiwan Warm Current; CC: Coastal Current (seasonal current with northward in summer and southward in winter); TC: Tsushima Current.

In this study, we tested the “pelagic seed bank” hypothesis for the initiation of *P. donghaiense* blooms in the ECS via introduction from the TWC front. Two surveys at different spatio-temporal scales were conducted in 2010 and 2011 (Fig. 1) with the objective of delimiting the TWC and the extent and abundance of *P. donghaiense* cells. The bloom pattern and development were examined in the surveys.

## Results

**Figure 2:**
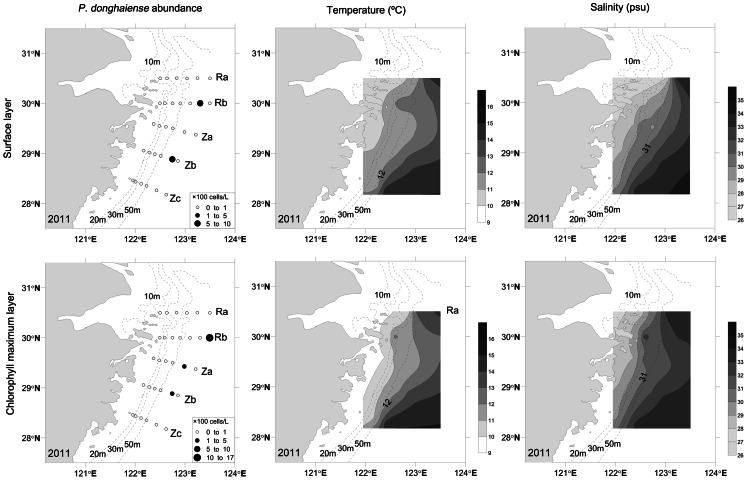
Distribution of *P. donghaiense* (left panels), temperature (middle panels) and salinity (right panels) in surface and chorophyll maximum layers in the ECS in the first cruise in 2011.

*P. donghaiense* cells were present above our detection limit of 2 cells/L at 38 of 41 stations during the 2010 comprehensive survey. The number of dinoflagellate species recorded varied from 1 to 9 (including *P. donghaiense*; mean±SD  =  3.61±1.95). The relative abundance of *P. donghaiense* versus total dinoflagellates varied from 28.60% to 100%, with an average of 96.80±9.12%. Few diatom species were recorded. In 2011, *P. donghaiense* cells were only present at stations Rb16, Rb17, Za6a and Zb12a in the first cruise but at all stations in the last cruise (Fig. 2).

**Figure 3:**
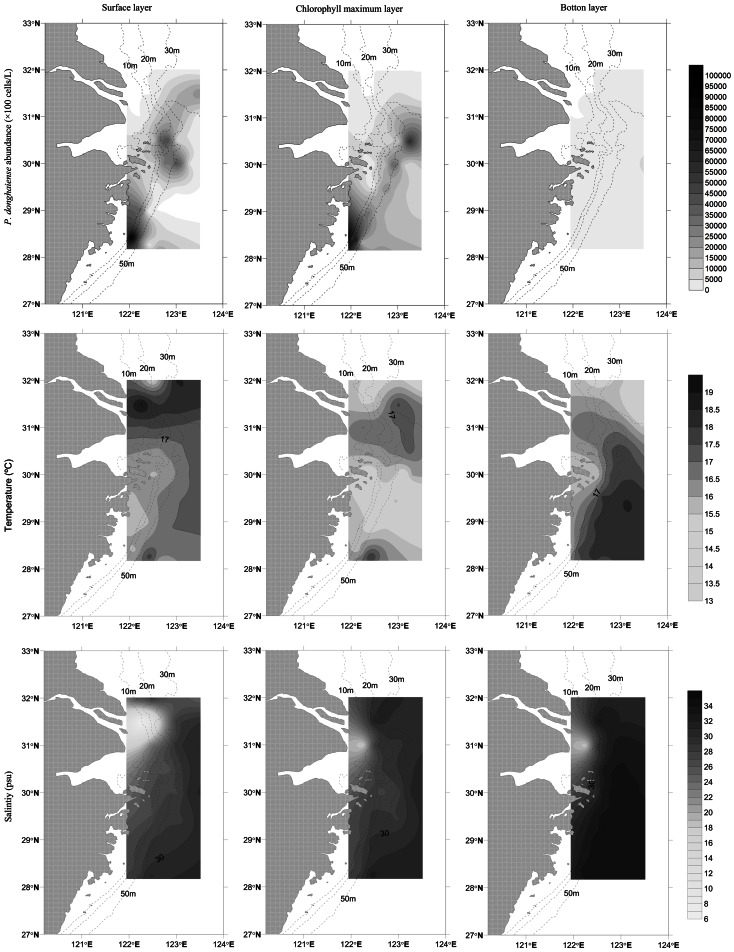
Distribution of *P. donghaiense* (top panels), temperature (middle panels) and salinity (bottom panels) in three different layers in the ECS in 2010.

**Figure 4:**
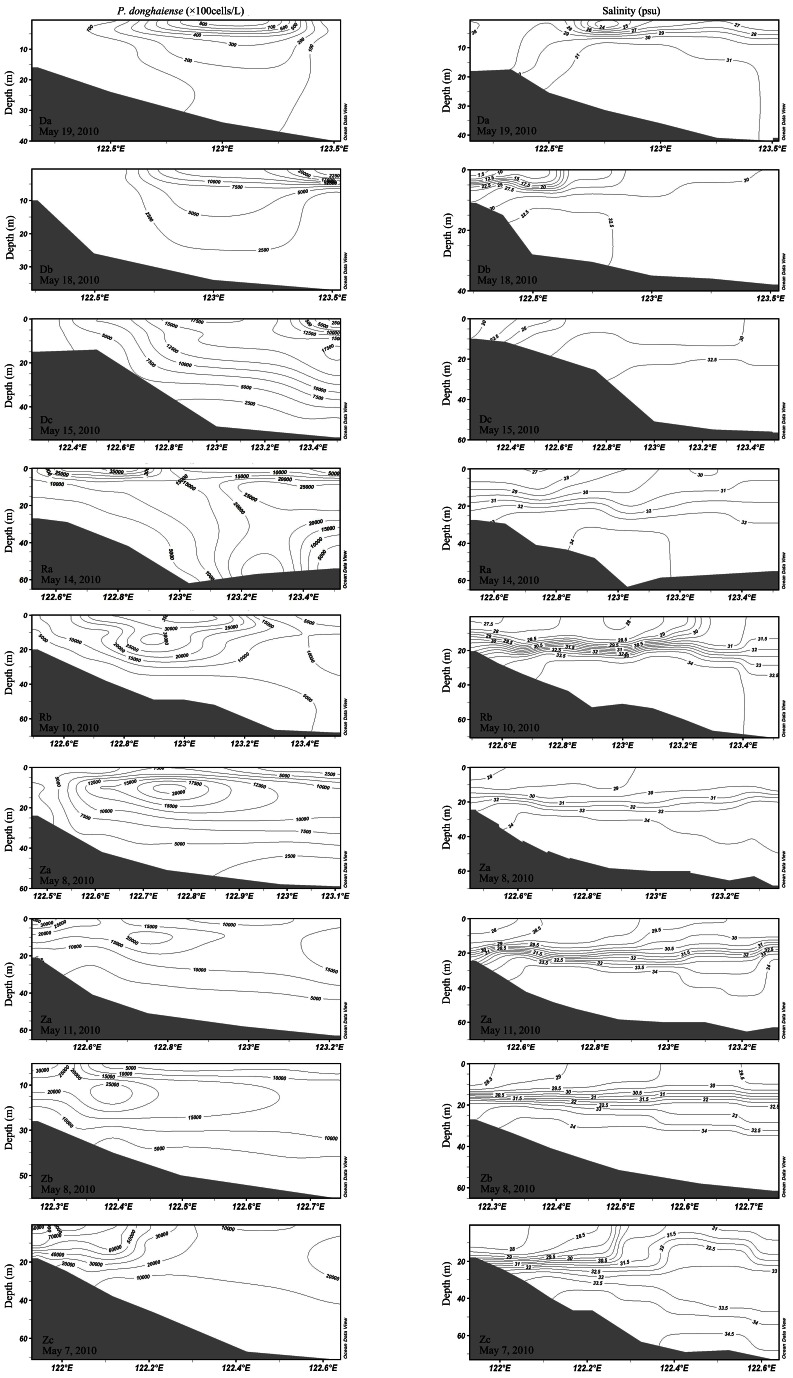
Vertical profiles of *P. donghaiense* abundance (left panels) and salinity (right panels) of nine transects (2 replicates for the transect Za) investigated in 2010.

**Figure 5:**
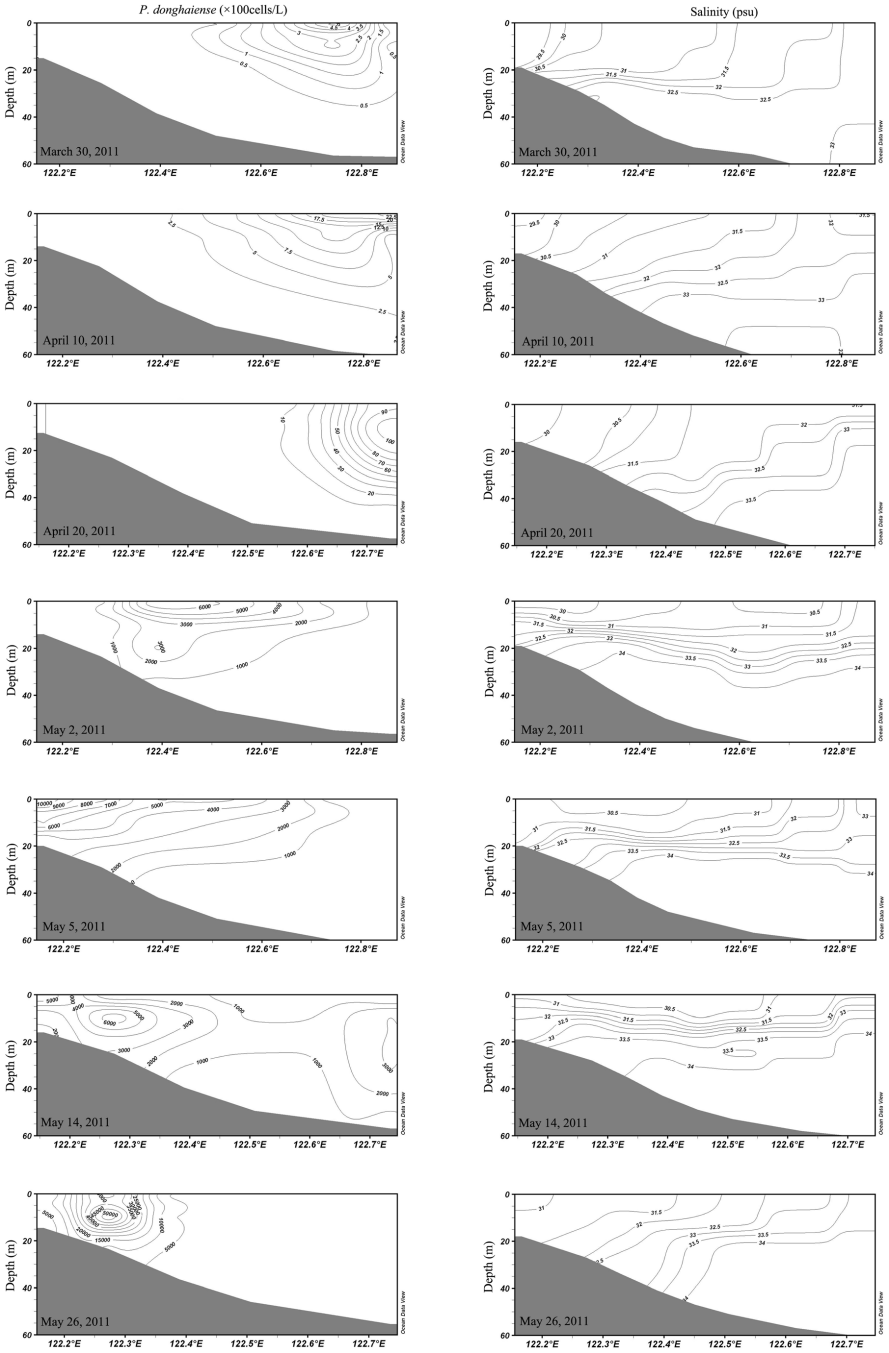
Vertical profiles of *P. donghaiense* abundance (left panels) and salinity (right panels) on the transect Zb in seven cruises in 2011.

*P. donghaiense* cell concentrations were highest along the upwelling zone near the coast (Figs. 3 and 4). At the surface, *P. donghaiense* concentrations were high at the Zhoushan Fishary Ground and in the area at 122°E, 28.5°N. In the chlorophyll maximum layer, a high abundance belt was observed around the 50 m isobath. In bottom layer, the abundance was low. The salinity distribution showed clearly that there was a fresh water plume in the Changjiang River estuary (Figs. 3 and 4). The pattern of *P. donghaiense* abundance was closely related with that of salinity, with rapid development of the *P. donghaiense* bloom during stratified conditions (Figs. 4 and 5). Moreover, the highest concentrations of *P. donghaiense* were located at the 10 m depth, near the TWC front on all transects (Figs. 4 and 5).

The highest concentrations of *P. donghaiense* formed early at the TWC front, and then spread inshore over the course of 2 months in 2011, coinciding with the incursion inshore of the TWC indicated by high salinity (Fig. 5). On the transect Zb, the cell abundance core was located at 122.8°E on March 30, 2011, displaying a low abundance of about 450 cells/L. It moved inshore to 122.1°E on May 26, 2011, exhibiting a high abundance of about 7×10^6^ cells/L. *P. donghaiense* bloom expanded rapidly in stratified water mass with relatively stable environment (Fig. 5). Interestingly, the core of maximum abundance of *P. donghaiense* bloom moved inshore from May 8–11, 2010 on the transect Za, while the water mass stratification did not change too much (Fig. 4).

## Discussion

Like most dinoflagellate blooms, large-scale *P. donghaiense* blooms in the ECS are affected by multiple factors including eutrophication from human activities [5]. In this study, we highlighted the role of the TWC front as a “pelagic seed bank” to inshore *P. donghaiense* blooms.

### The TWC front as a “pelagic seed bank”

The strong spatio-temporal match between *P. donghaiense* concentrations and the TWC is consistent to the hypothesis that the TWC front may serve as a seed bank to *P. donghaiense* blooms in the ECS (Figs. 2, 3, 4, 5). Algal cells aggregate along frontal zones because of the physical aggregation and barrier effect [6], [17], [18]. These aggregations are often evident as extraordinarily long bloom patches. Examples include a 300 km long patch of *Lingulodinium polyedrum* that has been observed in the California Current [19] and a 2000 km patch of *Akashiwo sanguineum* that has been observed within the Peru Current [19], [20]. Here, a similar belt pattern of *P. donghaiense* was recorded in two years, at the frontal zone between the Coastal Current (CC) and TWC (Figs. 2, 3, 4). The highest cell concentrations within these patches occurred at depths of approximately 10 m, consistent with the boundary of the TWC front in both survey years. These observations supported our hypothesis that the TWC front acts as a “pelagic seed bank” from which the *P. donghaiense* population could develop and expand to adjacent areas closer to the coast (Figs. 4 and 5).

We did not investigate whether *P. donghaiense* existed within the TWC here, but it was clear that *P. donghaiense* was present at the TWC front in early spring (Fig. 2). The high *P. donghaiense* concentration moved inshore with the TWC front after bloom initiation (Fig. 5). We suggested that the movement of the bloom inshore was caused by three factors: changes in the nearshore current flows, the swimming behavior of *P. donghaiense* and nutrient distribution.

With regard to changes in the nearshore current flows, the TWC front may play a role in driving *P. donghaiense* blooms inshore, similar to the transportation of *Cochlodinium polykrikoides* blooms by the Tsushima Current (TC) in the southern coast of Korea [18]. *P. donghaiense* bloom could develop inshore even in stable stratified water mass, for example, the movement of the bloom on the transect Za in 2010 (Fig. 4). This species has been recorded in the frontal zone of the TWC throughout the year during larger-scale surveys (unpublished data and [5]). *P. donghaiense* has also been recorded at higher latitudes (along the coasts of South Korea and Japan) where the TWC is entrained into the KC (Fig. 1 and [21]). However, *P. donghaiense* has not been recorded within the Yellow Sea, most likely because water mass in this region is dominated by fresh water flows from the Changjiang River and cold Yellow Sea coastal current [5].

The promotion of bloom development by water stratification is also partly attributable to the swimming behavior and chain formation of this species [1]. *P. donghaiense* has the characteristics cited as critical for pelagic seed bank species by Smayda (2002) when he proposed their existence, i.e., tolerates the turbulence and velocities in frontal systems, develop frontal zone populations and their successful advective transfer from these sites leads to blooms elsewhere [8].

### Nutrients

In the TWC, low nutrient concentrations likely limited *P. donghaiense* growth even though the temperature and salinity conditions were suitable for survival of this species [22]. In contrast, temperature was 8–12°C in the coastal zone during the winter, too low for *P. donghaiense* survival, but nutrient concentrations were relatively high due to enrichment from the Changjiang River and mixing via the CC [8], [23] and upwelling along the coastal zone [4], [9]. Therefore, *P. donghaiense* blooms would be triggered by increasing temperature within the coastal zone, especially since this species is more competitive in water with low phosphorus and excess nitrogen as a result of diatom blooms in early spring [3], [24].

Movement of the TWC also impacted the distribution of nutrient concentrations within the ECS, especially via the interaction of the TWC with the movement of fresh water along the coast. Nutrient concentrations decreased along transects from the coastal zone to offshore areas [25]. This nutrient gradient likely led to the stimulation of *P. donghaiense* blooms once they reached inshore areas. Nutrient concentrations are also enriched at depth along the TWC front because of the aggregation effect [8], which helps *P. donghaiense* easily inhabit this water column.

### Temperature and salinity

**Figure 6:**
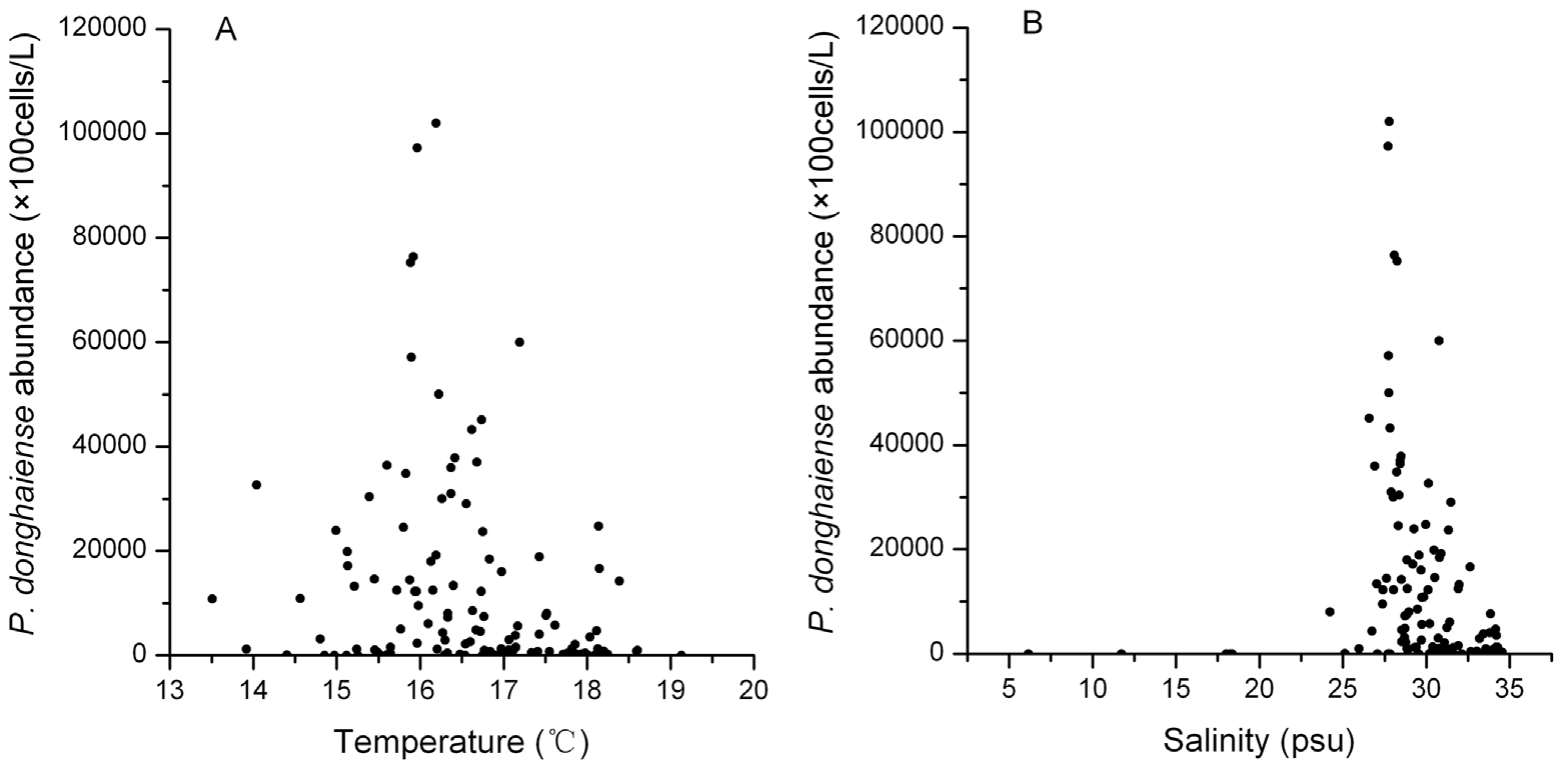
Relationships between *P. donghaiense* abundance and temperature (A) and salinity (B).

Temperature and salinity are both critical for the survival and distribution of *P. donghaiense* [15]. In winter, the low temperature of coastal water (<12°C) is unfavorable for growth or even survival by *P. donghaiense* [26]. In spring, a temperature and salinity window opens for *P. donghaiense* blooms through the movement of the TWC close to shore. Previous studies have reported that the optimal temperature range for *P. donghaiense* growth is between 20 and 27°C in cultures [15], and between 18.5 and 21.3°C *in situ* [27]. However in this study, the highest *P. donghaiense* concentration occurred at lower temperature, around 16°C (Fig. 6). This likely reflects the reality that many phytoplankton species live in suboptimal conditions in nature because of the trade-offs among many different environmental conditions [28]. In the ECS, nutrient concentrations were quite suitable for growth but the temperature was low within the coastal zone, while the temperature was optimal but the nutrient concentrations were low offshore.

*P. donghaiense* is likely to benefit from the combination of high temperature, high salinity and high nutrient water conditions within the interaction zone between the TWC and coastal fresh water. Once growth is initiated, *P. donghaiense* blooms may then expand to adjacent areas. This type of interaction may also disperse *P. donghaiense* into coastal areas without immediately triggering a bloom as was observed in the ECS during the 2005 season when springtime warming was delayed [29], [30]. In all cases – the 2005 season and the 2010 and 2011 seasons – the tight coupling of *P. donghaiense* with the TWC front in space and time suggests a pelagic seed bank strategy for the introduction of these cells into the nutrient rich coastal zone. The importance of such offshore source populations should be investigated further as they may play a significant role in the development of other dinoflagellate blooms.

## Materials and Methods

We declare that all necessary permits are obtained for the described field studies. No specific permissions are required for these locations. The location is not privately-owned or protected in any way. No endangered or protected species are involved in the field studies.

### Study area

Survey data was collected over the course of 10 research cruises. The first of these was conducted from May 7–24, 2010. The remaining nine cruises were conducted in 2011, from March 29 to April 2, April 9–10, April 19–20, April 28, May 2, May 4–7, May 13–15, May 22 and May 25–27, each along the selected transects of five transects (Ra, Rb, Za, Zb and Zc) between 28°N and 34°N (see Fig. 1). Most transects crossed the 20 m and 60 m isobaths in the coastal area of the ECS and the Yellow Sea (YS).

Within the study area, there is a seasonal switch whereby the Coastal Current (CC) flows southwestward in winter and northeastward in summer. Due to the winter-time direction of flow, the CC mixes with the fresh water from the Changjiang River during winter, transferring the nutrients from the estuary to the coastal region of Zhejiang Province [5]. In contrast, the flow of the TWC does not change seasonally (Fig. 1). The balance between these currents determines the movement of the TWC front [11]. Near 31.5°N, in the area offshore to the Changjiang River estuary, the fresh water of Changjiang River as well as the TWC turns from northeastward to eastward in late spring (Fig. 1) [5], [31].

### Sample collection

There were two types of stations: (1) comprehensive station in which both the hydrological and biological parameters were investigated; and (2) hydrological station in which only the hydrological parameter was investigated. At each comprehensive station, a CTD multiparameter sonde (SBE 19plus, Sea-Bird Electronics, Inc. USA) was used to profile from the sea surface to the bottom of the water column to determine the depth of chlorophyll maximum layer before sampling. Water samples were collected using 30 L Niskin bottles in surface layer, chlorophyll maximum layer and bottom layer. Extra sampling depths were added at selected stations. Water sample (500 ml) was transferred into 550 ml Polyethylene terephthalate (PET) bottles and was then fixed with 3–5% acidic Lugol's solution. At each station, environmental parameters such as temperature, salinity, density, turbulence, dissolved oxygen (DO), chlorophyll-a (Chla), irradiance and pH were recorded at every station at 0.5 m depth interval using the CTD.

Field samples were concentrated to 50 ml after sedimentation for more than 24 h in laboratory. And then, 1 ml subsample was transferred to 1 ml counting chamber (Sedgewick Rafter Counting Cell, PYSER-SGI LIMITED, UK) for observation using a light microscope (LEICA DMI 4000B, Germany) at 100× and 400× magnification. This step was repeated if the plankton abundance is low. Cell concentrations (cells/L) were estimated as 100×Cn/V, where Cn was the number of cells counted and V (ml) was the total volume of subsamples.

### Data analysis

The distributions of *P. donghaiense* and salinity were plotted with the Surfer software package (Version 8.0, 2002, Golden Software, Inc. USA) from biological observations taken at the comprehensive stations and hydrological observations from all stations.

Special emphasis was given to the transect Zb in our analysis because it was occupied during seven cruises and our hydrological data from these cruises showed substantial movement of the TWC front. Water salinity was used as a proxy for the TWC front because its variability was strongly correlated with water density in all of our surveys. Contour maps of *P. donghaiense* cells abundance and salinity on each transect were drawn using the Ocean Data View (ODV, 2007, http://odv.awi.de/).
